# The role of the community health delivery system in the health and well-being of justice-involved women: a narrative review

**DOI:** 10.1186/s40352-019-0092-y

**Published:** 2019-06-28

**Authors:** Sharla A. Smith, Glen P. Mays, Tracie C. Collins, Megha Ramaswamy

**Affiliations:** 1 Department of Preventive Medicine and Public Health, University of Kansas School of Medicine, 1010 N. Kansas Street, Wichita, KS 67214 USA; 2 Department of Health Management & Policy, College of Public Health, The University of Kentucky, 111 Washington Avenue #201, Lexington, KY 40536-0003 USA; 3 Department of Preventive Medicine & Public Health, University of Kansas School of Medicine, 1010 N. Kansas St., Ste 1406, Wichita, KS 67214-3199 USA; 4 Department of Preventive Medicine and Public Health, University of Kansas Medical Center, 3901 Rainbow Blvd, MS 1008, Kansas City, KS 66160 USA

**Keywords:** Justice-involved women, Community health delivery system, Justice, Social services, Healthcare, Incarceration

## Abstract

**Background:**

Over seven million imprisoned and jailed women are released into the community each year and many are ill-equipped to meet the challenges of re-integration. Upon release into their community, women are faced with uncertain barriers and challenges using community services to improve their health and well-being and reuniting with families. Few studies have identified and described the barriers of the community health delivery system (CHDS)- a complex set of social, justice, and healthcare organizations that provide community services aimed to improve the health and well-being (i.e. safety, health, the success of integration, and life satisfaction) of justice-involved women. We conducted a narrative review of peer-reviewed and gray literature to identify and describe the CHDS and the CHDS service delivery.

**Results:**

Peer-reviewed and gray literature (*n* = 82) describing the CHDS organizations’ missions, incentives, goals, and services were coded in three domains, justice, social, and healthcare, to examine their service delivery to justice-involved women and their efforts to improve the health and well-being of justice-involved women.

**Conclusions:**

We found that the CHDS is fragmented, identified gaps in knowledge about the CHDS that serves justice-involved women, and offer recommendations to reduce fragmentation and integrate service delivery aimed to improve the health and well-being of justice-involved women.

## Introduction

Women made up 7% of the total prison population at year-end 2017 (Bronson & Carson, 2017). The number of women incarcerated for more than 1 year increased by more than 700 prisoners in 2016 (Shinkfield & Graffam, 2009; Springer, 2010). In the United States, more than 11 million people are released from jails and prisons each year (Shinkfield & Graffam, 2009; Wolff, 2005). Women constitute nearly 16% of the correctional facilities population, with more than 75,000 in state prisons, close to 10,000 are in federal facilities, and 64,000 are in U.S. jails (Freudenberg, Daniels, Crum, Perkins, & Richie, 2008; Shinkfield & Graffam, 2009). Additionally, over one million women are under care custody and control (i.e. parole and probation) of correctional agencies (Freudenberg et al., 2008; Kajstura, 2019; Shinkfield & Graffam, 2009). About two-thirds of the incarcerated women will be under care custody and released into their communities, thus over 2 million women will reintegrate into their communities to continue to care for themselves and their families (Freudenberg et al., 2008; Shinkfield & Graffam, 2009).

Recent research has called for a special focus on how best to serve justice-involved women upon returning to their communities, the majority of whom will do so within days, weeks, or months of incarceration (La Vigne, Davies, Palmer, & Halberstadt, 2008). Upon returning to the community, justice-involved women will face significant barriers in obtaining housing, greater difficulty in obtaining and sustaining employment, less family support, and more substance abuse than men (La Vigne et al., 2008). They suffer from sexual abuse and mental illness and their experiences in the justice system may have led to re-traumatization (La Vigne et al., 2008). Additionally, many jails and prisons fail to provide women with basic hygiene and reproductive health needs adding additional burden on women during reintegration. (Visher, La Vigne, & Castro, 2003). Many justice-involved women return to low-income communities, where there are limited services and resources available to assist women in the re-integration process while meeting the requirements of probation and parole (Sprague, Scanlon, & Pantalone, 2017). Community re-integration is complex, as the women require a substantial number of social, justice, and healthcare services. In the first year after release, 35% of female prisoners were re-arrested and 14 per 100 women paroled return to jail because they fail to meet parole requirements (Alper, Durose, & Markman, 2018; Kaeble, 2018). Thus, justice-involved women must meet requirements which include regular meetings with their parole officer, stable housing, employment, and avoiding drugs and alcohol (Freudenberg et al., 2008; La Vigne et al., 2008; Richie, 2007; Visher et al., 2003) leaving them heavily dependent upon the community health delivery system (CHDS), which we define as a complex set of social, justice, and healthcare organizations that provide community services aimed to improve the health and well-being (i.e. safety, health, success of integration, and life satisfaction) of justice-involved women of justice-involved women. The CHDS is a complex system that lack of coordination to improve the health and well-being of justice-involved women.

Women face many barriers utilizing the CHDS due to a limited understanding of the provision of services, fragmented services, and accessibility (Freudenberg et al., 2008; La Vigne et al., 2008; Visher et al., 2003). The barriers faced by justice-involved women are compounded with the social stigma of having a criminal record, sexual abuse, higher rates of sexually transmitted diseases, and often minority status; therefore, women’s needs are often unmet and unknown by the CHDS (Glaze, 2009; Richie, 2007; Visher et al., 2003). The CHDS agencies vary widely in their missions, goals, and operations to improve the health and well-being of justice-involved women (Glaze, 2009; Richie, 2007; Visher et al., 2003). Identifying and defining the CHDS and understanding the role it plays in improving the health and well-being of justice-involved women is a vital step in improving CHDS services and programs delivery and population health.

The purpose of this narrative review is (1) identify the CHDS organizations, (2) summarize what is known about the CHDS, (3) understand the CHDS services that influence the health and well-being of justice-involved women, and (4) identify unanswered questions and the need for additional research. The system is referred to as the CHDS because they provide a complex set of social, justice, and health care services and activities that collectively increase the chances of successful integration in the community, reduce recidivism, and improve health and well-being. The CHDS organizations are not interdependent of each other. For this reason, the review reflects the systems-thinking lens that captures the ability to understand individual organizations and interconnections that influence the health and well-being of justice-involved women (Rosenblatt, 1993). The systems-thinking lens focuses on the interconnected set of elements and interconnections that are organized to achieve a function or purpose (Rosenblatt, 1993). While most re-entry research has solely focused on linkage to mental health and substance abuse treatment, housing, education, and employment, limited attention has been focused on the comprehensive system to address the cyclical problem (i.e. women cycle in and out of jail due to poverty and the inability to meet the obligations of their parole and probation) that women struggle to deal with due to justice-involvement (Freudenberg et al., 2008; Glaze, 2009; La Vigne et al., 2008; Richie, 2007; Rosenblatt, 1993; Shinkfield & Graffam, 2009; Visher et al., 2003; Wolff, 2005). Therefore, this article presents findings from a narrative literature review focused on the diverse CHDS agencies and services recognizing that most research focuses on individual health care linkages and there is limited knowledge of the CHDS as a whole.

## Methods

### Search strategy

Due to the paucity of information on the CHDS to answer the research question, we conducted a narrative review of peer-reviewed and gray literature that discusses and describes the science and knowledge of community resources using the systems- thinking lens to identify and summarize what is known about the CHDS and enhance the understanding of the CHDS on the health and well-being of justice-involved women. The research questions used to identify the literature was “What are the community resources aimed to promote the health and well-being of justice-involved women?”. Which organizations provide the identified community resources? A number of PubMed, PsycINFO, and web of sci searches were performed, and additional websites were reviewed. To be confident the new evidence was not missed, two websites, OATD and ProQuest of dissertations, and current website review were conducted to review conference abstracts. To ensure that we identified relevant evidence from a variety of disciplines (e.g., criminal justice, public health, health care, social services, social work, and law and policy), we searched using mesh terms and databases described in Appendix 2. We identified 139 peer-reviewed and gray literature (i.e. unpublished research such as websites, conference abstracts, dissertations, and reports) published and updated between 1980 and 2017. We focused on literature between 1980 and 2017 because the number of women in United States prisons increased by 700% since 1980 and approximately 9 million women are released into the community each year, leading researchers to focus on the CHDS aimed to improve the health and well-being of justice-involved women (Morash, Kashy, Smith, & Cobbina, 2014; The Sentencing Project, 2017;). The primary focus of this review is to identify and understand the CHDS for justice-involved women (i.e. post-incarcerated, paroled, and probation), excluding other studies (i.e. qualitative and quantitative studies on incarcerated women) limited on post-release information. The review was restricted to studies related to community re-entry and community resources.

All peer-reviewed and gray literature (*n* = 82) were considered if they met the following criteria: (1) community reintegration for post-incarcerated women, (2) post-release interventions for incarcerated women, (3) parole and probation for women, (4) community services for post-incarcerated women, and (5) healthcare for post-incarcerated women. Gray literature such as websites, conference abstracts, dissertations, and reports was included. Peer-reviewed and gray literature that did not meet these criteria were excluded.

### Data extractions

The 82 peer-reviewed and gray literature were reviewed and coded using the three domains and subdomains. Domains were defined based on the literature’s objectives, keywords, and results. Literature was grouped into domains and counted.

## Results

Tables 2, 3 and 4 summarizes the literature included in the review and described in Appendix 3. The overarching conclusion from all the literature reviewed was that justice-involved women utilized several CHDS organizations in the following domains: 1) justice, 2) social, and 3) healthcare Appendix 1. The justice organizations are agencies that institute practices to uphold social control, deter and mitigate crime, and sanction those who violate the law while providing or referring women to health and social services to improve their health and well-being (Dias & da Silva Junior, 2016; State, County, Municipal Courts, 2017; Swavola, Riley, & Subramanian, 2016). The justice agencies included are parole and probation departments, police departments, and courts including court officials-judges, administrators, prosecutors, deputies, and public and private defenders, prison and jails. Social organizations are agencies that provide a range of public services to improve the health and well-being of justice-involved women, their families, and their communities (Colbert & Durand, 2016; Huebner, DeJong, & Cobbina, 2010; Parsons & Warner-Robbins, 2002; Swavola et al., 2016; Yamatani & Spjeldnes, 2011). The social service agencies included are housing and urban development, department of children and families, welfare, workforce, substance abuse treatment centers, mental health, food pantries, local health departments, Medicaid, and faith-based organizations. Healthcare organizations are public and private agencies that provide healthcare services to justice-involved women to prevent, alleviate, and cure illness and injuries (Colbert & Durand, 2016; Huebner et al., 2010; Parsons & Warner-Robbins, 2002; Swavola et al., 2016; Yamatani & Spjeldnes, 2011). Healthcare agencies included are substance use and mental health treatment, community health centers, and hospitals. The purpose of this narrative review is (1) identify these CHDS organizations, (2) summarize what is known about these CHDS, (3) understand the CHDS services that influence the health and well-being of justice-involved women, and (4) identify unanswered questions and the need for additional research.

### CHDS stakeholders

#### Domain one: justice organizations

The community health delivery system should be established to encompass the agencies justice-involved women have initial and continuous contact with and to set conditions in which they must comply in order to gain successful re-entry into their communities. The justice organizations encompass a complex number of organizations and vary widely in service delivery, resources, missions, goals, and incentives. The four main components of the justice system that respond to crime and victimization in communities are (1) law enforcement, (2) the courts, (3) institutional corrections facilities (e.g., jails, prisons), and (4) community corrections programs (probation, parole). Although the organizations within this system are well known by name and the processes but the resources, missions, goals, and incentives geared to meet the unmet needs and expectations of justice-involved women are unknown (Bell, Perez, Goodman, & Dutton, 2011; Clear, 2007; Cobbina, Morash, Kashy, & Smith, 2014; Covington, 2001; Covington, 2007; Daly, 1987; Golder, Hall, & Logan, 2014; Judging Science, 1999; Lam & Harcourt, 2003; Morash et al., 2014; Opsal, 2009; Petersilia, 2001; Schram, Koons-Witt, Williams, & McShane, 2006; Smith & Visher, 1981; The Sentencing Project, 2007; Women in the Criminal Justice System: Briefing Sheets, 2007; Zeoli, Rivera, Sullivan, & Kubiak, 2013). The unknown relationships, services, and programs highlight the needs to identify justice system organizations and understand their service delivery, resources, missions, goals, and incentives.

### Courts

The literature revealed that the initial point of contact in determining the future health and well-being of justice-involved women are courts (Bell et al., 2011; Clear, 2007; Covington, 2001; Covington, 2007; Daly, 1987; Judging Science, 1999; Lam & Harcourt, 2003; Morash et al., 2014; Opsal, 2009; Schram et al., 2006; The Sentencing Project, 2007; Women in the Criminal Justice System: Briefing Sheets, 2007; Zeoli et al., 2013). There are many different types of courts at the federal, state, county, and municipal levels. Our review revealed the vital courts for justice-involved women are the municipal and county courts that determine the terms and conditions of both parole and probation since almost 60% of women are convicted of non-violent crimes (i.e. drug and property) and re-enter their communities within days or months after their conviction (Bell et al., 2011; Judging Science, 1999; The Sentencing Project, 2007; Women in the Criminal Justice System: Briefing Sheets, 2007). The municipal court hears most justice-involved women cases because the majority of women are convicted of non-violent crimes (Bell et al., 2011; Women in the Criminal Justice System: Briefing Sheets, 2007). In 2014, the rate of women convicted of non-violent drug crimes were 210.7 and 364.7 for property crimes (Carson, 2018). Women convicted of assault crimes encounter the county court, which hears two different types of cases, civil and criminal. In 2014, the rate of women convicted of assault crimes were 188.5 (Carson, 2018). The goal of the municipal and county courts are to determine whether the women accused of the crime are guilty and determine the punishment for the crime which also includes substance and drug abuse treatment (Bell et al., 2011; Daly, 1987; Judging Science, 1999; The Sentencing Project, 2007; Women in the Criminal Justice System: Briefing Sheets, 2007). Additionally, approximately 60% of justice-involved women are mothers and therefore, encounter family court at the same time they encounter municipal or county court (Bell et al., 2011; National Resource Center on Justice Involved Women, 2016; Women in the Criminal Justice System: Briefing Sheets, 2007). The family court decides the degree to which parents will have physical and legal custody of, or parenting time (also termed visitation) with, the child and whether they regain custody after criminal justice involvement (Salem, Nyamathi, Idemundia, Slaughter, & Ames, 2013). Although justice-involved women encounter at least two of the justice organizations at the same time, the limited knowledge of the organizations’ missions, incentives, and goals may become interwoven with the direct and collateral consequences-homelessness, drug and sexual abuse, mental health, inability to seek health care, lack of health insurance, and unemployment, of justice-involvement directly linked to their criminal activity (Smith & Visher, 1981).

### Community corrections programs

Most justice-involved women are released “conditionally” or sentenced based on conditional provisions of parole or probation (Covington, 2007; Kruttschnitt, 2010; Petersilia, 2001). The parole population continues to grow, increasing by 0.5%, from 870,500 persons at year-end 2015 to 874,800 at year-end 2016 (Kaeble, 2018). In 2016, over 1.1 million women were supervised in the community under care custody- (probation or parole)- (Shinkfield & Graffam, 2009; Wolff, 2005). In 2016, 25% of justice-involved women were on parole and assigned a parole officer that supervises their ability to adhere to the terms and conditions of their conditional parole or probation (Cobbina et al., 2014; Golder et al., 2014; Kaeble, 2018; Kruttschnitt, 2010). Parole is a period of conditional supervised release in the community followed by state or federal prison (Kaeble, 2018). The mission of the parole office is to promote public safety and strive for justice and fairness while assuring the terms and conditions ordered by the court are followed to prevent recidivism (Kruttschnitt, 2010; The United States Department of Justice, 2018). These terms include but are not limited to living within state and county lines, meeting regularly with a parole officer, submitting to random drug and alcohol testing, and providing proof of residence and employment (Cobbina et al., 2014; Kruttschnitt, 2010). Unlike parole that is granted after an offender serves a portion of their prison sentence, probation may be granted as an alternative to a jail sentence or a combined sentence involving incarceration followed by a period of community supervision (Cobbina et al., 2014; Golder et al., 2014; Kaeble, 2018; Kruttschnitt, 2010). The justice-involved women on probation may live freely in the community but must abide by certain conditions of probation for a period of time and regularly report to a probation officer (Cobbina et al., 2014). The general conditions of probation are similar to those of parole living where directed, participating in court required services and programs including mental and substance abuse treatment, submitting to random drug or alcohol test, housing, and maintaining employment (Cobbina et al., 2014; Kruttschnitt, 2010).

### Law enforcement and community policing

The increase in justice-involved women can be linked to changes in law enforcement practices targeting minority neighborhoods (National Resource Center on Justice Involved Women, 2016). The criminal justice process starts at the point of contact with a law enforcement officer and once released on parole or on community supervision, women have daily contact with law enforcement. However, limited research has focused on the larger population of women who have not been incarcerated and are not on probation or parole but had previous justice-involvement (Cobbina et al., 2014; Covington, 2007). These justice-involved women have daily contact with law enforcement officers because these individuals patrol the communities in which they work, live, and play (Covington, 2007). Law enforcement has a mission to protect and safeguard the lives and property of the people they serve (Covington, 2007; Morash et al., 2014). Some law enforcement departments have set up diversion programs, designed to refer justice-involved women whose behavior may indicate trauma, substance abuse, or mental health to treatment (Worden & McLean, 2018). Law Enforcement Assisted Diversion is an approach used by law enforcement to redirect low-level offenders engaged in drug or prostitution activity to community-based services, instead of jail and prosecution (Worden & McLean, 2018). In 2013, over 2700 communities had implemented crisis and non-crisis intervention teams, which involve specially trained officers and mental health professional responding to crises together or law enforcement assessments and direct referrals to community treatment and services. These approaches often help make treatment more accessible and avert the short and long-term disruption to women’s lives from short stays in jail and the collateral consequences of a conviction. However, the approach lacks the ability to connect women to other vital services that improve their health and well-being, such as housing, health care, and employment.

### Jails

Jails are confinement facilities operated under the authority of a sheriff, police chief, city or county administrator that primarily hold incarcerated person including women who are charged with committing a criminal offense or awaiting the resolution of their cases for short-term periods (Covington, 2001; Petersilia, 2001; Smith & Visher, 1981). In 2016, jails housed over 113,000 women awaiting trial, sentencing, or transfer to prison; parole or probation violators, and those sentenced to less than one year (Kajstura & Marigeon, 2015; Zeng, 2019). Due to the short-term stays of women in jails, limited research has focused on the post-release and the impact on the health and well-being (Covington, 2001; Petersilia, 2001; Smith & Visher, 1981). As a result, jails experience a high turnover and affect a far greater swath of the population (Kajstura, 2019). About one-fifth of justice-involved women often cycle in and out of jails, not because they commit a new crime, but rather they break rules of their parole and probation such as failing a drug test or missing a scheduled appointment (Kajstura, 2019). The high turnover and shorter stays make screening and health care challenging for the unique needs (i.e. reproductive care, sexually transmitted diseases, family planning, and sexual and drug abuse) of this population (Kajstura, 2019). Reversing the cycling in and out of jails can be difficult addressing the unmet unique needs of this population (Kajstura & Marigeon, 2015).

### Prison

Prisons are facilities in which women are confined and denied the authority of the state for many years (Petersilia, 2001). In 2016, women made up 7% of the total national prison population. From 2015 to 2016, the number of women sentenced to more than 1 year in state or federal prison increased by 700; the rise in the number of women in prison can be traced to changes in the state and national drug policies (Carson, 2018). Changes in law enforcement practices and post-conviction barriers to reentry uniquely affect women (Lam & Harcourt, 2003; Petersilia, 2001; Smith & Visher, 1981). Women prisoners are vulnerable and it is, therefore, necessary to pay particular attention to preventing, monitoring, and treating women-specific health problems, while in prison and upon release (Ramirez, 2016; Willging, Nicdao, Trott, & Kellett, 2015) of the one-quarter of women released from prison fail within 6 months (i.e., have an arrest for a new crime), one-third fail within a year and two-thirds fail five years after release (Willging et al., 2015). Not all prison provide eligible women with trauma treatment, pregnancy programs, and other needed health care services. Prisons often fail to meet the basic and complex needs of women by limiting and charging women for basic needs such as menstrual and feminine products, soap, toothpaste, toothbrushes, and shampoo (Huebner et al., 2010; Smith & Visher, 1981). The inability of the prison to meet the women’s basic and health care needs has negative implications on the health and well-being of the 21% of women released from prison in 2016 (Alper et al., 2018). Prisons lack coordination with community health providers needed to continue care for women upon their release. Recent literature on the impact of imprisonment has attempted to estimate the impact of imprisonment on post-release experiences and circumstances (Bell et al., 2011; Clark, Dolan, & Farabee, 2017; Dias & da Silva Junior, 2016; Glaze, 2009; Morash et al., 2014; O’Brien, 2007; Shinkfield & Graffam, 2009) but is limited in the prison’s role to coordinate with community organizations to ensure smooth transitions and successful reintegration for women.

#### Domain two: social service organizations

The complexities of re-establishing life after criminal involvement include securing housing, formal identification, finding a job, and re-applying for other social services (Department of Children and Family, 2009; Kruttschnitt, 2010). Additionally, the majority of justice-involved women have children and because the children have needs of their own, women must have contact with social service agencies that have conflicting or otherwise incompatible goals and values than some social services (McCarty, Falk, Aussenberg, & Carpenter, 2012; Smith & Visher, 1981).

Several social service agencies also have stake and interest in the preventive recidivism and can be valuable in efforts to provide vital service and program to a vulnerable population (Jason, Salina, & Ram, 2016; Salem et al., 2013). These agencies are housing and urban development, department of children and families, welfare, workforce, substance abuse treatment centers, mental health, food pantries, health departments, Medicaid, and faith-based organizations. The Department of Housing and Urban Development (HUD), children and family services, welfare, and workforce operate under multiple authorities and missions to provide a variety of resources, while their missions and incentives are very different these agencies aim to provide safe and affordable services and are often considered second chance services for vulnerable populations (Braithwaite, Treadwell, & Arriola, 2008; Jason et al., 2016).

### Housing

Decent and affordable housing is critical to the well-being of women released from jail or prison. Without safe and stable housing, justice-involved women are directly in the path of violence, sex work, drugs, and other high-risk life choices (Department of Children and Family, 2009; Jason et al., 2016). Research suggests housing is the most important social service for these populations as housing determines whether justice-involved women have access to other social services (Dekeseredy, Alvi, & Tomaszewski, 2003). While some justice-involved women live with family upon release or while on probation, some may not have a family willing to house them (Jason et al., 2016). As a result, justice-involved women are almost 10 times more likely to be homeless than the general public (Jason et al., 2016.; Dekeseredy et al., 2003; Department of Children and Family, 2009). The Department of Housing and Urban Development (HUD) is working to strengthen the housing market for all persons and provides justice-involved women hope for safe and affordable housing (Jason et al., 2016). Despite HUD’s mission to increase the availability of affordable, decent, and accessible housing for all residents, justice-involved women are three times more likely to spend years on the housing authority waiting list than the general public and often rejected by a string of property owners based on a prior conviction and are ineligible based on U.S. housing policies (Jason et al., 2016).

### Department of Children and Family Services

The Department of Children and Family (DCF) is vital to justice-involved women regaining custody of their children. The goal of the DCF is creating a safe and stable environment for children and reunite children with their parents (Clark et al., 2017; Warners-Robbins & Parsons, 2010). In 2016, 1 in 8 incarcerated parents lost their parental rights regardless of the seriousness of their offenses (Hager & Flagg, 2018). Women prisoners’ children are five times more likely than male prisoners children to be placed in foster care (Hager & Flagg, 2018). It is difficult for justice-involved women to advocate for their children during this time and even harder reuniting with them upon release or after probation (Warners-Robbins & Parsons, 2010). Most women who give birth while incarcerated have to hand over their baby to a family member or friends (Warners-Robbins & Parsons, 2010). However, if no one can help, the baby goes to DCF. Reuniting with your children often requires working closely with a DCF casework and adhering to supervised visitation with children (Warners-Robbins & Parsons, 2010). Although women complete the requirements within a few months, they may wait for years to be reunited with their children (Warners-Robbins & Parsons, 2010). It has been acknowledged that women who do not have a good rapport with DCF or additional help from other local organizations are less likely to be reunited with their children (O’Brien, 2007; Warners-Robbins & Parsons, 2010). Yet, little is known about relations between DCF and justice-involved women’s health and well-being due to the lack of matched child protective services and incarceration and post-incarceration data (Berger et al., 2016). A better understanding of this relationship can improve services directed to families interacting with DCF and the criminal justice systems.

### Temporary assistance for needy families

Justice-involved women, like other women and parents in the community, need money and employment to support their families and be a part of mainstream society (Holtfreter & Morash, 2003; Sprague et al., 2017). Unlike many social services organizations, Temporary Assistance for Needy Families (TANF) and workforce have coordinated efforts wherein TANF is a substitutional income program that assists women in their transition to the workforce and workforce identifies employment opportunities (Holtfreter & Morash, 2003; Sprague et al., 2017). TANF and Food Stamps assist justice-involved women with cash assistance and food during their transition into the workforce (Holtfreter & Morash, 2003; Sprague et al., 2017). However, TANF is the most difficult program to access—it entails lengthy application processes and upfront work activities that create barriers for justice-involved women (Berger, Cancian, Cuesta, & Noyes, 2016; Holtfreter & Morash, 2003; Huebner et al., 2010). Numerous federal laws have imposed a lifetime ban on welfare services such as TANF and food stamps regardless of efforts of rehabilitation efforts (Holtfreter & Morash, 2003). Although some states have opted out of the ban, many states require drug tests thus justice-involved women have difficulties complying with the work requirements of these programs, which often leads them to discontinue court-ordered programs and other social service requirements (Holtfreter & Morash, 2003). As a result, women may be required to choose between meeting the requirements of their parole and probation or doing what is required to receive these services. These barriers serve as deterrents for accessing TANF and limit women’s ability to use other social services, meet the obligations of their parole and probation, live stable lives, and improve their health and well-being.

### Medicaid

Medicaid is the largest single payer of direct medical services for vulnerable populations (DiPietro & Klingenmaier, 2013; Enard & Ganelin, 2013; Hirsch, 1994; Richie, 2007). Medicaid agencies aim to provide health and long-term care insurance to vulnerable populations (DiPietro & Klingenmaier, 2013). Despite this intention, many states have failed to expand Medicaid to ensure this vulnerable population receives Medicaid upon their release (DiPietro & Klingenmaier, 2013; Enard & Ganelin, 2013; Hirsch, 1994). Although research shows that providing individuals with access to the needed health care services upon release reduces the likelihood of recidivism, 19 states terminate rather than suspend or reclassify Medicaid for women entering the justice system (DiPietro & Klingenmaier, 2013; Enard & Ganelin, 2013; Hirsch, 1994). Suspending and prescreening justice-involved women who are eligible to receive Medicaid services will allow women to gain quicker access to mental health treatment, prescribed medicines, and other needed care upon release as well as reduce paperwork for the state (Richie, 2007). The termination of Medicaid benefits creates barriers to the receipt of health care upon release (DiPietro & Klingenmaier, 2013; Enard & Ganelin, 2013; Hirsch, 1994). Upon release, many women lack the needed documentation (i.e. identification and physical address) to obtain Medicaid and have limited transportation to make required Medicaid and doctor appointments and therefore forego their healthcare needs (Richie, 2007). Although Medicaid offers transportation services and mileage reimbursement, justice-involved women may have limited knowledge of services that could reduce the barriers to seeking healthcare (DiPietro & Klingenmaier, 2013).

### Faith-based organizations

Members of the faith-based community seek to live out their religious and spiritual faith through various activities to make their communities safer places to live (Huebner et al., 2010; Parsons & Warner-Robbins, 2002). Spiritual teams and individuals within these organizations participate in efforts that focus on breaking the cycle of crime and incarceration to improve the quality of their neighborhoods (Huebner et al., 2010; Parsons & Warner-Robbins, 2002; Swavola et al., 2016). To that end, they have found creative ways to respond to the unmet needs of justice-involved women in their communities (Huebner et al., 2010; Swavola et al., 2016). Not only do they build relationships with women prior to their release from jail or prison, but they also work closely with parole and probation officers to assist women with unmet needs post-release (Colbert, Sekula, FAU- Zoucha, Zoucha, & Cohen, 2013; Huebner et al., 2010). Faith communities have provided various aftercare ministries that include showers, food pantries, shelters, financial assistance, and referrals to other services and resources (Colbert et al., 2013; Huebner et al., 2010). Faith-based communities earn justice-involved women’s trust because of the work done with the women prior to their release from jail or prison and with parole and probation officers to meet justice-involved women unmet needs (Huebner et al., 2010; Swavola et al., 2016). However, a limitation of the faith-based organization’s services is the requirement to participate in prayer and religious activities as a stipulation of assistance, which justice-involved women are often forced to comply with (Huebner et al., 2010). Another limitation is that these faith-based services are not guaranteed services – they are voluntary, highly community specific, and individualized (Huebner et al., 2010; Parsons & Warner-Robbins, 2002; Swavola et al., 2016).

### Local health departments

Local health departments (LHD) are the backbone of the local public health system with a mission and goal to improve health, wellness, safety, and quality of life in the community (Huebner et al., 2010; Nargiso, Kuo, Zlotnick, & Johnson, 2014; Parsons & Warner-Robbins, 2002; Smith & Visher, 1981). LHDs help create and maintain conditions in communities that support healthier choices, lead efforts to prevent and reduce chronic illnesses, and protect children and families from infectious diseases (Nargiso et al., 2014; Parsons & Warner-Robbins, 2002; Smith & Visher, 1981). They play a central role in providing essential public health services in communities (Abbott, Magin, Lujic, & Hu, 2017). As a part of their role and mission, they provide health promotion programs and services to vulnerable populations such as justice-involved women (Abbott, Magin, Lujic, & Hu, 2017). Justice-involved women are more likely to suffer from chronic and communicable diseases like HIV, Hepatitis C, and sexually transmitted infections than women in the general population (Kelly, Hunter, Daily, & Ramaswamy, 2017). In addition, they require pre-and post-natal and family planning services because they are at high risk for unintended pregnancies and substance abuse upon release (Kelly et al., 2017). These services are often obtained at an LHD due to their ability to provide services at no or little cost (Kelly et al., 2017). Not only do LHDs provide services to justice-involved women but they also provide services to their children such as women, infant, and children services, immunizations, and physicals (Abbott, Magin, & Davison, 2017).

### Community re-integration programs

Community reentry programs have been proven to help reduce recidivism rates and improve the health and well-being (i.e. safety, success of integration, and referrals and linkage to social and health services) of justice-involved women (Bloom, 2006; Eggers, Munoz, Sciulli, & Crist, 2006; Rich et al., 2001; Richie, Freudenberg, & Page, 2001). Many justice-involved women have difficulty integrating into the community and meeting the conditions of parole due to their limited ability to find a job, adequate housing, and attain photo identification (Richie et al., 2001). Some re-entry programs provide the opportunity for inmates to gain basic living skills on how to successfully transition back into the community after release (Help for Felons, 2017). Re-entry programs offer a comprehensive list of services such as short-term housing, food, clothing, job assistance (i.e. placement, training, and career development), medical and dental care, substance abuse treatment, and mental health services (Richie et al., 2001). Forty-six states have community re-entry programs that vary from faith-based programs to comprehensive community re-entry programs that provide education on the department of corrections, job training and placement, and family reunification (Help for Felons, 2017; TheLlion Heart Foundation, 2018). For example, a well-documented New York reentry program, Health Link, is designed to assist drug-using jailed women in New York City to return to their communities, reduce drug use and HIV risk behavior, and avoid rearrests by working directly with women in the jail and after release and by addressing the community conditions that hamper successful reintegration. (Richie et al., 2001). Re-entry programs are broadly defined as organizations that serve individuals released from the criminal justice system into the community thus may vary in services and resources limiting their ability to provide a comprehensive approach that may improve the health and well-being of justice-involved women (Richie et al., 2001). The development of programs that engage women in navigating the CHDS through referrals and work directly with other CHDS agencies may help women change the conditions of their lives by reducing drug use, improving their health, avoiding dangerous relations, and improving their well-being.

#### Domain three: healthcare organizations

Two weeks following release, women prisoners are at a 12 fold increased risk of death, which highlights the need for timely linkages to medical care and preventive services during community reentry (Kelly et al., 2017). However, for women leaving prison or jail, there are roadblocks that reduce or eliminate the ability to seek or continue needed services such as alcohol and drug treatment and other healthcare treatment to successfully reintegrate into their communities (Kelly et al., 2017; Rogers, 2015). Recent studies have shown that justice-involved women have a myriad of health issues compounded by unique circumstances in the re-entry into their communities (Abbott, Magin, & Davison, 2017; Erin & Ost, 2007). Additionally, justice-involved women are less likely to receive routine care (i.e. well-women exams and screenings) and may not seek health care treatment because of barriers such as the lack of health coverage, cost, limited knowledge of the health system, and the demands to meet the obligations of probation, parole, and family (Abbott, Magin, & Davison, 2017; Erin & Ost, 2007). These barriers often lead to the increased use of the hospital’s emergency departments (ED) for chronic and minor health care (Bandara, Huskamp, & Riedel, 2015). However, substance abuse and mental health treatment and community health centers (CHC) particularly safety-net clinics remove the financial barrier to seeking health care (Hirsch, 1994; Richie, 2007). Despite the availability of free health care services in multiple communities, women face competing priorities such as housing, finding employment, re-establishing relationships, and attending regular parole meetings, which may delay the receipt of medical care (Abbott, Magin, & Davison, 2017; Kelly et al., 2017); Ryan, Pagel, & Smali, 2016).

### Substance use and mental health

Drug and alcohol problems and critical mental health issues have reached epidemic proportions for justice-involved women (Jason et al., 2016; Lyons, 2010; Nargiso et al., 2014; Sprague et al., 2017). Documented underlying behaviors that cause women to have justice involvement is a history of substance abuse and mental illness (Lyons, 2010; Nargiso et al., 2014). An estimated 80% of individuals released from prison in the United States each year have a substance use disorder, or chronic medical or psychiatric condition (Guyer, Serafi, Bachrach, & Gould, 2019). In 2012, 65.8% of women prisoners and 67.9% of women in jails reported a history of mental health problems (Bronson & Berzosky, 2017). Many individuals utilize substances to cope with mental illness or physical health, which often creates the co-occurrence of substance abuse with persisting mental health problems (Lyons, 2010; Nargiso et al., 2014). Although both substance abuse and mental health treatments are vital to the recovery of women with a history of drug abuse, justice-involved women are often referred to only substance abuse treatment (Jason et al., 2016; Lyons, 2010;Nargiso et al., 2014 ; Roth et al., 2012). The central mission of substance abuse treatment centers is to help individuals with substance abuse and underlying mental health needs in order to improve lives (Nargiso et al., 2014). Effective substance abuse programs work with clients to broaden their range of response to various types of behaviors and needs, enhancing their coping and decision-making skills with an empowerment model to help women achieve self-sufficiency (Jason et al., 2016; Lyons, 2010). In addition, effective therapeutic approaches are multidimensional and deal with specific women’s health and well-being, including chemical dependency, domestic violence, sexual abuse, pregnancy and parenting, relationships, and gender bias (Nargiso et al., 2014; Roth et al., 2012). Though substance abuse treatment is most often provided to justice-involved women, the barriers to receiving treatment and care from both substance and mental health treatment services are often ignored (Lyons, 2010; Roth et al., 2012). The receipt of both substance and mental health treatment services has the potential to reduce recidivism.

### Community health care centers

Correctional facilities provide health care for justice-involved women while in jail or prison (Abbott, Magin, & Davison, 2017; Rogers, 2015). However, there is little to no coordination between the correctional facilities’ health care services and community health centers (Abbott, Magin, & Davison, 2017; Rogers, 2015). Community health centers are well positioned to provide preventive and follow-up health care to justice-involved women (Abbott, Magin, & Davison, 2017; Rogers, 2015). CHCs have a mission to provide high-quality, accessible medical, dental and behavioral health services to individuals and families at an affordable cost or free (Rogers, 2015). The CHC is less likely to meet the needs of vulnerable populations due to limited knowledge about the provision of services and lack of coordination with correctional facilities (Rogers, 2015).

### Emergency rooms

The ED serves as an entry point for inpatient admissions and is a common setting for acute care (Bandara et al., 2015). Several studies have reported that women with a history of incarceration were more likely to have poor health outcomes and use EDs as a regular source of care (Bandara et al., 2015). A recent study found that justice-involved women had a higher proportion of frequent ED visits (27.2% vs 9.4%) than women with no criminal justice contact (Bandara et al., 2015). A history of incarceration among women has been associated with a higher prevalence of infectious diseases, chronic diseases, and a higher risk of death (Bandara et al., 2015; Bracken, Hilliard, McCuller, & Harawa, 2015; Erin & Ost, 2007). The poor health of justice-involved women has led to the overutilization of emergency rooms resulting in limited primary care engagement (Bandara et al., 2015). Research suggests that primary care engagement improves the health and well-being of justice-involved women and has a positive association with stable housing and satisfaction of housing (Bracken et al., 2015). ED physicians do not provide follow-up service or have the staff on hand to deal with these multiple health conditions: substance abuse, physical and sexual abuse, and mental health (Bandara et al., 2015). Justice-involved women that utilize ED as usual source of care often have an untreated or undiagnosed medical condition, which in some cases can lead to continuing risky behaviors that may threaten their health and well-being (Erin & Ost, 2007).

## Discussion

Over the past 30 years, America has expanded the use of prisons and jails and increased criminal punishment for women (Morash et al., 2014; Smith & Visher, 1981). The expansion of punishment penetrates deeply into the fabrics of women’s lives inside and outside correctional institutions (Visher & Travis, 2003; Morash et al., 2014; Smith & Visher, 1981). For example, justice-involved women are forced to navigate a complex community health delivery system, including justice, social, and healthcare organizations, to meet the requirements of their parole and probation and reintegrate into their community. The requirements of parole and probation are often aligned with the priorities of obtaining housing, employment, substance abuse treatment, and required appointments with probation and parole officers (Bridgman, 2002; Broner, Lang, & Behler, 2009; Dekeseredy et al., 2003; Department of Children and Family, 2009; Jason et al., 2016; Kruttschnitt, 2010; Kurlychek, Brame, & Bushway, 2006; Metraux & Culhane, 2004; Salem et al., 2013; Walter, Viglione, & Tillyer, 2017; Why Punish the Children?, 1993). Although many CHDS organization directors and staff do not feel they have a role in improving the health and well-being of justice-involved women, navigating the CHDS often creates barriers to seeking needed health care and leads to missed opportunities to redirect women toward healthier, stable, and more productive lives in the community (Rogers, 2015). Yet little is known about the comprehensive CHDS justice-involved women must navigate to reintegrate into their communities. Limited research has focused on identifying and understanding the CHDS and its impact on the health and well-being of justice-involved women.

Although the CHDS for justice-involved women has been identified in our review, the identification of the organizations is only the first step to identifying the gaps and uncertainties in the programs and services for justice-involved women. Our review suggests that the CHDS is a complex fragmented system made up of individual organizations that have limited alignment in missions and independent practices that spawn inefficient allocation of resources for justice-involved women. The review also suggests that the remaining uncertainties that justice-involved women confront daily in their attempt to navigate the CHDS are unknown (Morash et al., 2014; Rogers, 2015; Smith & Visher, 1981). Additionally, the gaps in the provision of CHDS services and programs for justice-involved women is also unknown. Expanding the research to focus on the integration of the CHDS and its barriers and facilitators for justice-involved women will require several key approaches to research.

First, research is needed to identify opportunities to integrate CHDS services and programs. The growth of the incarcerated female population and health and human services funding cuts have contributed to the fragmentation of the CHDS by increasing workloads limiting the ability to meet justice-involved women needs with limited community resources, budgets, and evidence-based practices (Shinkfield & Graffam, 2009; Springer, 2010). Predictably, solo (i.e. parole and probation officers) or small single (i.e. faith-based organizations, re-integration programs, and substance abuse treatment centers) have dominated the landscape, with a variation in services, costs, referrals and follow-up, and low accountability. The limited research that identifies the justice organizations as contributors to justice-involved women health and well-being contributes to the CHDS fragmentation, thereby interposing an inherent disconnect between social and health care organizations interest and the interest of justice-involved women (Cobbina et al., 2014; Golder et al., 2014; Petersilia, 2001; Smith & Visher, 1981). The increasing incarceration rate, cycling, and the unique constellation of health and social problems of incarcerated women suggest that justice-involved women require services, programs, and care from multiple CHDS providers in multiple settings and accountability for seeking these services (Shinkfield & Graffam, 2009; Springer, 2010). The opposite of a fragmented CHDS is a coordinated, integrated CHDS similar to integrated healthcare and public health delivery system (Enthoven, 2009; Strange, 2009). The CHDS may improve the alignment of missions and practices by implementing a tracking system that can manage referrals or transitions and allows organizations to close-the-loop in the system. An integrated CHDS is an organized, coordinated, and collaborative system that (1) links various social services providers, (2) coordinated efforts with justice organizations, (3) is accountable for and has a system in place to manage and improve the health and well-being of justice-involved women (Enthoven, 2009; Strange, 2009).

Second, research is needed to identify the barriers and facilitators of each CHDS and the role they play in justice-involved women’s ability to seek preventive health care. Numerous known and unknown barriers and facilitators may hinder justice-involved women’s ability to seek needed care. Although it is well known that a large majority of justice-involved women have risky behaviors and receive health care while incarcerated, little is known about how to best engage justice-involved women in their health care within the context of re-entry and criminal justice supervision (i.e. parole and probation) (Covington, 2007; Erin & Ost, 2007; Freudenberg et al., 2008; Rogers, 2015; Rosenblatt, 1993). Justice-involved women are unlikely to seek the needed preventive sexual health care or practice healthy sexual behaviors often encouraged by primary care physicians because most seek their care in an ED (Cobbina et al., 2014; Covington, 2007; Institute of Medicine, 2002; Ramaswamy, Upadhyayula, Chan, Rhodes, & Leonardo, 2015). Identifying barriers and facilitators will not only enhance the women’s ability to seek needed health care but enhance the knowledge of CHDS providers and may lead to strategic approaches to eliminate barriers and enhance facilitators.

Third, research should focus on strengthening the CHDS and documenting the impact of the CHDS on the health and well-being of women. Due to limited state budgets and capacity in CHDS, many have limited resources to meet the needs of vulnerable populations (Broner et al., 2009; Dias & da Silva Junior, 2016; Richie, 2007). The CHDS need health promotion plans that target systematic and vulnerable population problems and resources to develop strategies to intervene and address problems (Celinska & Siegel, 2010; Cobbina et al., 2014; Covington, 2001; Erin & Ost, 2007; Ramaswamy et al., 2015; Rogers, 2015). The recommended approaches to strengthen the CHDS may improve its infrastructure and capacity to effectively meet the needs of justice-involved women through service provisions, integration of services, collaborations, and policies. Additionally, expanding the target-substance abuse and mental health of existing diversion programs to address the overall health and well-being of women may enhance the capacity of the CHDS to integrate services and programs (Rogers, 2015).

Fourth, research needs to focus on the CHDS policies and procedures and the role they play in creating barriers for justice-involved women to seek preventive health care. Policies are often the result of choice by autonomous decision makers at each phase of the justice system whose actions are based on their limited knowledge of the CHDS and its impact on the health and well-being of justice-involved women (Hirsch, 1994; Jason et al., 2016; Parsons & Warner-Robbins, 2002).

Finally, our review indicated that the current body of literature on CHDS for justice-involved women are limited and demonstrates the need to examine the system comprehensively (Rosenblatt, 1993: Judging science, 1999; Institute of Medicine, 2002). Although a full examination of the comprehensive CHDS for justice-involved women is beyond the scope of this review, our findings demonstrate that many CHDS organizations have services and programs for justice-involved women and may recognize their role in justice-involved women reintegrating into the community (Richie et al., 2001; Rogers, 2015; Shinkfield & Graffam, 2009). Thus, research should expand to examine the CHDS current role in reintegration and the integration of CHDS services and programs to ensure that justice-involved women receive needed resources and guidance after release. These efforts may make the difference between recidivism, successful transition to the community, and improving the health and well-being of justice-involved women.

## Conclusions

The Institute of Medicine (IOM) recommended that closer collaboration and integration between governmental public health agencies and the health care delivery system may enhance the capacities of both to improve population health (Institute of Medicine., 2002). Our review represents the first literature review to identify the comprehensive CHDS that may collectively enhance the capacity of the CHDS to integrate services and programs for the purpose of reducing recidivism and improving justice-involved women’s health and well-being.

Some limitations of the review should be noted. First, our review does include a number of assessments of CHDS programs and services under development and not in peer-reviewed and gray literature. While we reviewed a number of conference abstracts in gray literature, additional literature on the CHDS may be underrepresented in both gray and peer-reviewed sources used in our review.

Despite the limitations, the results of our review help to fill the gap in the current literature by identifying CHDS organizations and pointing to targeted areas of future research to examine the programs, services, and impact of CHDS on the health and well-being of justice-involved women. Continued efforts toward documenting existing CHDS programs, understanding the mechanisms through which CHDS organizations improve the health and well-being of women, as well as increasing the integration of CHDS services and programs are an important means of strengthening the scientific knowledge base of health services research, programs, and policies.

## Figures and Tables

**Fig. 1:**
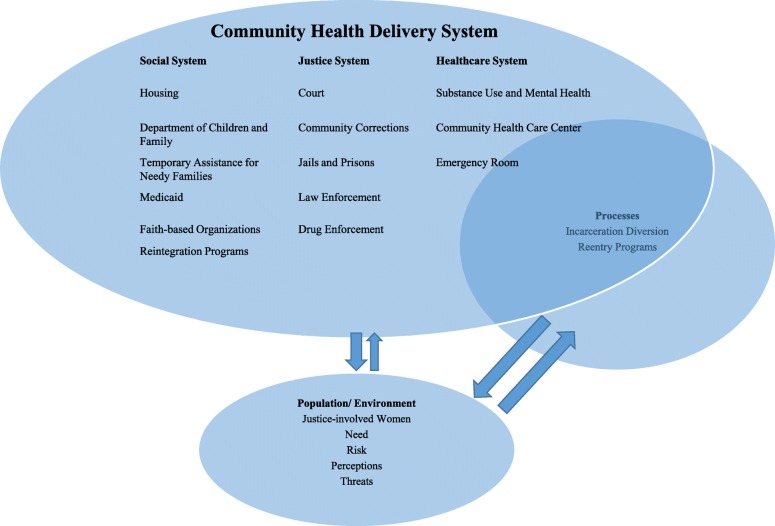
Community health delivery system

## Data Availability

The submitted publication is a literature review and the data is available via many search engines and have been included in the Appendix.

## Appendix 2

Search words and medical subject headings (MeSH) terms.

**Table 1:** Search words and medical subject headings (MeSH) terms

Search Words/MESH Term (1980-2017)	No. of Articles Found
Incarcerated women	919
Post Incarcerated women	96
Incarcerated women and health	38
Post incarcerated women and social work	10
Social services and vulnerable populations	15
Social welfare and post incarcerated women	10
Social justice and incarcerated women	3
Justice system and post incarcerated women	35
Justice-involved women	70
Marginalized women	678
Child services and incarcerated women	38
Department of human services	17
Healthcare and Incarcerated women	209
Criminal system and women	459

## Appendix 3

Literature review tables.

**Table 2:** Domain one: justice organizations literature review

Source	Study Description	Purpose	Results
Justice			
Alper, M., Durose, M. R.,Markman, J. (2018). 2018 Update on Prisoner Recidivism: A 9-Year Follow-up Period (2005–2014). U.S. Department of Justice, Bureau of Justice Statistics. Retrieved from https://www.bjs.gov/content/pub/pdf/18upr9yfup0514.pdf.	A report that examines the post-release offending patterns of former prisoners and their involvement in criminal activity both within and outside of the 10 state where they were imprisoned.	The Bureau of Justice Statistics analyzed the offending patterns of 67,966 prisoners who were randomly sampled to represent the 401,288 state prisoners released in 2005 Year after release in 30 states.	Excluding probation and parole violations, 82.4% of prisoners released in 30 states in 2005 were arrested within 9 years.
Bell ME, Perez S, Goodman LA, Dutton MA. Battered Women Perceptions of Civil and Criminal Court Helpfulness: The Role of Court Outcome and Process. *Violence Against Women* 2011; 17:71–88.	A mixed methods study that utilized-3 questions using a Likert-type scale and eight open- ended interview questions with women who sought help from civil, criminal court and/or shelter.	To reveal general categories of factors contributing to helpfulness of the court system as a whole from the perspective of women who have experienced intimate partner violence.	For quantitative items overall, most women felt positive about their experience. Qualitative responses revealed two broad categories: court outcome issues and most responses were re-court process issues.
Carson, E. A. (2018). Prisoners in 2016. Retrieved from https://www.bjs.gov/content/pub/pdf/p16.pdf.	A report of the National Prisoner Statistics program, which collects annual data from state departments of corrections and the Federal Bureau of Prisons on prisoner counts, characteristics, admissions, releases, and prison capacity.	To provide prisoner counts and the percentage change in population of prisoners and jails.	The number of prisoners under state and federal jurisdiction at year-end 2016 (1,506,800) was a 7% decrease (down 108,700 prisoners) from 2009 when the U.S. prison population peaked. Females made up 7% of the total national prison population at year-end 2016, an increase of more than 100 prisoners from 2015.
Clear TR. Imprisoning Communities: How Mass Incarceration Makes Disadvantaged Neighborhoods Worse. Oxford, England: Oxford University Press, 2007.	A book that provides a thoughtful and provocative look at how “mass incarceration” has increased crime and other social ills in troubled neighborhoods.	To provide evidence that demonstrates the effects of imprisonment on many neighborhoods.	Clear calls for sentencing reform designed at ending mass incarceration, proposing fewer and shorter prison sentences in favor of community justice.
Covington SS. Women and the Criminal Justice System. *Women’s Health Issues*17:180–182.	The editorial highlights that women offenders are disproportionately women of color with health and mental health needs that require the development of comprehensive, coordinated services.	Highlights the need for correctional facilities and community health care providers to work together and create a meaningful system of care.	N/A
Daly K. Discrimination in the Criminal Courts: Family, Gender, and the Problem of Equal Treatment*. *Social Forces* 1987; 66:152–175.	The quantitative study evaluated 2004 defendants and analyzed the disposition of cases and sentence received by sex, marriage status, and dependents.	To identify what explains the variability of socioeconomic effects across dif**f**erent court outcomes.	Case severity, charge severity, type of the offense charged, and prior record for both men and women are treated dif**f**erently based on their familial ties and responsibilities to others.
Freudenberg N. Adverse Effects of US Jail and Prison Policies on the Health and Well-Being of Women of Color. *Am J Public Health* 2002; 92:1895–1899 (Freudenberg, 2002).	Commentary	To examines correctional processes that do little to nothing to address complex health, social, and economic issues that are only compounded by incarceration.	Recommends there is a need to study the more fundamental causes underlying multiple disparities in multiple conditions. Interventions that are gender- specific by way of policy change targeting social processes will be key.
Glaze LE. Correctional populations in the United States, 2011. 2012. Washington, D.C., U.S. Department of Justice, Bureau of Justice Statistics. 11–13- 2017.	A Report of correctional population in 2011.	The report provides a summary data on the total population under the supervision of the adult correctional systems and highlights significant changes in the components of the population.	The annual change in the total correctional population during 2008 was calculated as the sum of four components: the changes in the probation (up 36,446) and parole (up 6992) populations within 2008, the change in the jail population (up 5359), or the dif**f**erence between the June 30 population in 2007 and 2008; and the change in the custody prison population (up 4967), or the dif**f**erence between the December 31 populations in 2007 and 2008.
Incarcerated Women and Girls. The Sentencing Project. 2015. 11-15-2017.	The Sentencing Project is a leader in changing the way Americans think about crime and punishment.	To highlight the profound change in the involvement of women within the criminal justice system	There has been a 716% increase in the number of women incarcerated in the US since 1980.
Kajstura A, Marigeon R. States of Women’s Incarceration: The Global Context. 2015. 10-1-2017.	An online report that documents how women fare in the world’s carceral landscape.	The report compares the incarceration rates for women of each U.S. state with the equivalent rates for countries around the world.	Currently, prisons and jails in the U.S. confine approximately 206,000 women (at a rate of 127 per 100,000). Women should be a mainstay of any state policy discussions on the economic and effective use of incarceration if we hope to incarcerate fewer women.
Kajstura, A. (2019). Women’s Mass Incarceration: The Whole Pie 2019. Retrieved from https://www.prisonpolicy.org/reports/pie2018women.html.	A report on the systems of confinement.	To provide a detailed look at where and why people are locked up in the U.S., and dispels some modern myths to focus attention on the real drivers of mass incarceration.	The data makes it clear that ending the war on drugs will not alone end mass incarceration, though the federal government and some states have taken an important step by reducing the number of people incarcerated for drug offenses.
Judging Science. Scientific Knowledge and the Federal Courts. Nat Med 1999; 5:979-980.	Book Review.	The book provides a guidance for judges tat include 1. Hypothesis set forth is testable; 2. Theory or technique has been peer reviewed 3. Practical rate of error must be considered. 4. Method/theory has gained general acceptance. The purpose is to review the guidance and its impact.	There is a double edge to the suggested requirements. Having judges that are “scientifically literate” seems like a gain to ferret marginal science out but can be a barrier to admitting legitimate evidence in courtroom by field experts.
Lyons T. Recovery Capital, Drug Policy and The Cycle of Incarceration. *Practicing Anthropology* 2010;32:41–44.	A qualitative study utilizing the ethnographic and ecological perspective on prisoner re-entry.	Applies lessons learned from the ethnographic literature to identify the elements of the Treatment Alternatives for Safe Communities that are succeeding in keeping clients out of prison.	An ethnographic and ecological perspective on prisoner’s re-entry demonstrates the limitations of programs, which solely target the individual and ignore the community and policy context.
National Resource Center on Justice Involved Women (2016). Fact Sheet on Justice Involved Women in 2016. Retrieved from https://cjinvolvedwomen.org/wp-content/uploads/2016/06/Fact-Sheet.pdf .	A report of statistics on justice- involved women.	To provide some basic facts about justice-involved women, and how they are different from their male counterparts.	Women are more likely than men to commit property crimes such as larceny-theft and fraud, and are also more likely to commit drug offenses, including drug possession and trafficking and are less likely than men to have been convicted of a violent crime.
Opsal TD. Women on Parole: Understanding the Impact of Surveillance. *Women & Criminal Justice* 2009;19:306–328.	Qualitative interviews of 43 women and their perceptions of the parole system.	To look at gender-specific approaches of re-entry processes that facilitate positive re-entry outcomes.	The current parole model and the process produce feelings of fear, anxiety, and powerlessness.
Opsal TD. Women on Parole: Understanding the Impact of Surveillance. *Women & Criminal Justice* 2009;19:306–328.	A qualitative study that evaluated justice-involved women who explained how they perceived parole as a tool intended to monitor their actions as opposed to assist them in getting back on their feet.	To explore how a group of 43 women re-entering their communities via parole.	The findings demonstrate how parole produces feelings of fear, anxiety, and powerlessness in individuals and how this af**f**ects women newly released from prison who are working to regain control over their own lives.
Richie BE, Freudenberg N, Page J. Reintegrating women leaving jail into urban communities: A description of a model program. *Journal of Urban Health* 2001; 78:290–303.	A randomized control study of 700 inmates - one cohort received in jail services, seven other cohorts received in-jail services, and one-year post- release case management to evaluate which had a greater impact on.	Incarceration policies are inextricably linked with living conditions in low- income urban communities. Jails are unique points of opportunity as a place for intervention implementation and leverage women’s receptiveness to that intervention. Interventions must be done at every level that includes empowerment approaches and community organizingstrategies.	Women receiving the full Health Link services had a rearrests rate that was 21% lower than jail services only group (38% vs. 59%).
Schram PJ, Koons-Witt BA, Williams FP, McShane MD. Supervision Strategies and Approaches for Female Parolees: Examining the Link Between Unmet Needs and Parolee Outcome. *Crime & Delinquency* 2006; 52:450–471	Case-control study of 546 female parolees from a western state who have just finished their parole terms or who had been terminated from parole between Nov1997- Feb1998.	To examine the types of needs identified at intake from a sample of 546 female parolees.	65.2% of women were parole failures after 1-year release. 38% of the women were assessed for substance abuse needs. Of those identified as having a need, only 48% received some type of treatment.
Smith DA, Visher CA. Street- Level Justice: Situational Determinants of Police Arrest Decisions. 1981; 29:167–177.	Retrospective cohort pro-bit analysis of 742 police-citizen encounters from 1977 involving 24 police depts. To measure strength of association b/t arrest and location, bystander presence, race of the suspect, and sex of suspect.	To estimate the direct effects of situational variables on the arrest probabilities are important to understanding but only approximates the complexity of the arrest process.	Results provide the following: police do respond to the gravity of an offense, police are more likely to apply more formal sanctions against Blacks, the presence of bystanders increases the likelihood of arrest, and citizen input is reflected in police behavior.
State, County, Municipal Courts., 2017. 10–18-2016.	NA	A description of the state, county, and municipal courts.	N/A
Swavola E, Riley K, Subramani an R. Overlooked: Women and Jails in an Era of Reform. *Report* [serial online] 2016;1–48 Available from Vera Institute of Justice. Accessed July 1, 2017.	Report	To offer a portrait of women in jail, explore how jail can deepen the societal disadvantages they face, and provide insight into what drives women’s incarceration and ways to reverse the trend.	A foundation for reform exists and can potentially set the stage for further, well- crafted programs and practices to stem the flow of women cycling through the nation’s local jails. First, however, justice systems— both small and large—and community stakeholders must commit to bring women into the discussion.
The Sentencing Project. Women in The Criminal Justice System: An Overview. 2007. 10–20-2017.	A brief documenting the gender implications of changes that have occurred over the last 20 years within the criminal justice system.	To highlight the rate of women’s incarceration calls for a critical evaluation of the social impact of our nation’s increasing reliance on correctional facilities to deal with women’s involvement in the crime.	There is an increasing need for further consideration of the nature of women’s involvement in crime in order to respond appropriately to the personal and structural causes of their criminal behavior rather than relying solely on punitive responses.
The United States Department of Justice. U.S. Parole Commission. Retrieved from https://www.justice.gov/uspc.			
Zeng, Z. (2019). Jail Inmates in 2017. Retrieved from https://www.bjs.gov/content/pub/pdf/ji17.pdf.	A report of the nationally representative survey of county or city jail jurisdictions and regional jails in the country.	To track changes in the number and characteristics of local jail inmates nationwide, jail inmate turnover, jail capacity, and space usage by other authorities.	From 2005 to 2017, the male incarceration rate decreased by 12%, from 448 to 394 per 100,000 male residents, while the female incarceration rate grew by 10%, from 63 to 69 per 100,000 female residents.

**Table 3:** Domain two: social organizations literature review

Source	Study Description	Purpose	Results
Social			
Abbott P, Magin P, Lujic S, Hu W. Supporting continuity of care between prison and the community for women in prison: a medical record review. Aust Health Rev. 2017 Jul; 41 (3): 268–276	A retrospective review of medical records of 212 medical records of women who were in for at least 6 weeks or more and released from correctional facilities.	To examine health information transfer and continuity of care arrangements between prison and community health care providers for women in prison.	At release, continuity of care arrangements and health information transfer to general practitioners were usually linked to formal pre-release healthcare linkage programs. At release, only 20% of records had evidence of such continuity of care at release.
Abbott PA, Magin P, Davison J, Hu W. Medical homelessness and candidacy: women transiting between prison and community health care. Int J Equity Health. 2017; 16: 130.	A qualitative study including interviews of 69 incarcerated women 40 pre-release and 29 post-release in Australia.	To examine the ways in which women in contact with the prison system experience access to health care, particularly those with histories of problematic substance misuse.	Long wait lists impeded the ability for prisoners to get health needs met. The dual stigma of being a prisoner and drug user lead to provider adjudication and dismissal of women’s concerns are not being legitimate.
Bandara, S.N., Huskamp, H.A., & Riedel, L.E. (2015). Leveraging the affordable care act to enroll justice-involved population in Medicaid: state and local efforts. *Health Affairs (Millwood), 34*, 20,044–2051	A quantitative survey was administered to collect information on whether the programs’ jurisdictions used any of four specific policy approaches to facilitate Medicaid enrollment.	To characterize the national landscape of programs enrolling criminal justice–involved populations in Medicaid as of January 2015	The authors identified sixty-four programs that enrolled justice-involved individuals in Medicaid during detention, incarceration, or the release process. Fifty-seven of these were in states that had chosen to expand Medicaid, and seven were programs that targeted disabled populations and operated in states that had not expanded Medicaid as of January 2015.
Berger, L.M., Cancian, M., Cuesta, L. & Noyes, J. (2016). Families at the Intersection of the Criminal Justice and Child Protective Services Systems. Ann Am Acad Pol Soc Sci. 2016 May; 665 (1): 171–194.	A longitudinal data analysis of 2013 Multi-Sample Person File to describe intergenerational and intragenerational overlap in the two systems.	To examine both intergenerational and intragenerational overlap in incarceration and child protective services (CPS) involvement.	8% of all children experiencing a screened-in report had a parent in state prison at some point during 12 months following the report. Over 15% of adults in prison had one or more CPS- involved children and just almost 6% had children in OHP.
Broner N, Lang M, Behler SA. The Effect of Homelessness, Housing Type, Functioning, and Community Reintegration Supports on Mental Health Court Completion and Recidivism. *Journal of Dual Diagnosis* 2009;5:323–356.	A quantitative study that analyzes the self-reported quality of life and social support, chart diagnosis, and administrative housing, services, and criminal justice data collected from 589 Bronx Mental Health Court participants for 12 months following diversion.	To examine whether community stability indicators predict program completion and delay re-arrest for homeless versus non-homeless mental health court participants.	Mental health court was generally beneficial to mental health court participants. However, for those previously homeless, functioning and social support may play a unique and interconnected role in court graduations, whereas general life satisfaction may be a better indicator for program completion for non-homeless individuals.
Bronson, J. & Berzosky, M. (2017). Indicators of Mental Health Problems Reported by Prisoners and Jail Inmates, 2011–12. U.S. Department of Justice. Retrieved from https://www.bjs.gov/content/pub/pdf/imhprpji1112.pdf.	A report on the mental health problems among state and federal prisoners and local jail inmates.	To examine the prevalence of the two mental health indicators by different time periods, demographics, criminal justice history, and current offenses. Among state and federal prisoners and local jail inmates.	The percentage of prisoners who met the threshold for serious psychological distress (14%) was more than three times that of adults in the standardized total U.S. general population (5%) or those in the standardized general U.S. population with no criminal involvement in the past year (4%)
Celinska K, Siegel JA. Mothers in Trouble: Coping With Actual or Pending Separation From Children due to Incarceration. *The Prison Journal* 2010;90:447–474.	74 semi-structured interviews with mothers before trial and during incarceration to document coping strategies employed to deal with potential or actual separation from their children.	To document coping strategies employed to deal with potential or actual separation of women from their children.	Seven strategies emerge being a good mother, mothering from prison, role redefinition, disassociation from prisoner identity, self-transformation, planning and preparation, and self-blame. The findings show that mothers used multiple strategies and tended to employ emotion-focused and adaptive coping techniques.
Clark N, Dolan K, Farabee D. Public health alternatives to incarceration for drug offenders. EMHJ 2017, 23 No. 3.	A review that examines alternative approaches to drug offenses internationally as it recognizes the high costs and negative returns on incarceration.	To identify the public health alternatives to high costs and negative returns associated with imprisonment.	30 countries reformed drug policies to permit forms of decriminalization allowing for fewer people in prison, reducing criminal justice costs, redirecting law enforcement towards serious and violent crimes, minimizing social exclusion. A systematic review of drug courts found that participants
Cobbina JE, Morash M, Kashy DA, Smith SW. Race, Neighborhood Danger, and Coping Strategies Among Female Probationers and Parolees. *Race and Justice* 2014;4:3–28.	A three-part mixed methods study using 402 drug- involved women on probation or parole recidivism.	To examine whether residential segregation & related restriction of Blacks to areas of concentrated disadvantage is apparent without correctional population and explore self-directed efforts to cope with neighborhood crime & views of strategy effectiveness.	Black women reported more types of criminal activity in neighborhoods than white women. Black women lived in census tracts with higher disadvantages & lower affluence, stability than did white. Of the 295 women that described their neighborhood as unsafe, 86% had strategies women used to avoid offending.
Dekeseredy WS, Alvi S, Tomaszewski EA. Perceived collective efficacy and women’s victimization in public housing. *Criminal Justice* 2003; 3:5–27.	Quantitative analysis of 325 quality of neighborhood life survey questionnaires completed at 6 public housing estates and distributed to 1200 households.	To conceptualize why women in neighborhoods with poverty and limited employment report higher rates of victimization.	35% respondents reported hardly ever get together with neighbors, 78% do not belong to any social clubs, 42% said drugs are easy to access in residence, 25% reported being targeted for violence, and 27% reported having been victims of at least one of the four types of public/sexual harassment
Dishon-Brown AF, Golder S FAU - Renn T, Renn TF, Winham KF, Higgins GE FAU, Logan TK. Childhood Victimization, Attachment, Coping, and Substance Use Among Victimized Women on Probation and Parole.	A quantitative study of multivariate regression models of data on 406 women on parole or probation.	To investigate the relationship between attachment, coping, childhood victimization, substance use, and IPV among 406 victimized women on probation/parole.	Childhood sexual victimization and negative coping were significant in all analysis.
Freudenberg N, Daniels J, Crum M, Perkins T, Richie BE. Coming Home From Jail: The Social and Health Consequences of Community Reentry for Women, Male Adolescents, and Their Families and Communities. *Am J Public Health 2008;98: S191- S202.*	Randomized trial and evaluation of a case management and social support intervention designed to reduce drug use and rearrests among incarcerated women and male adolescents in New York City.	To describe the living conditions of people released from urban jails; to examine individual, community, and policy factors associated with post- release drug use and criminal activity; and to consider the implications of these findings for public policies related to reentry into the community from jail.	For men (mean age = 17) having a job, health insurance and marijuana use were associated with lower rearrests rates. Previous arrests, substance abuse, & having many peers regularly attending school/work were all more likely of being rearrested. For women: factors associated with rearrests were drug/ETO related social problems since release, homelessness, and previous arrest.
Golder S, Hall MT, Logan TK et al. Substance Use Among Victimized Women on Probation and Parole. *Substance Use & Misuse* 2014; 49:435–447.	The study examined among 406 victimized women on probation and parole in an urban community from 2010 to 2013.	To examine substance use among women on parole or probation.	93% of women reported lifetime use of an illicit substance, whereas 58% and 45% reported the use of at least one illicit substance in the past 2 years and 12 months, respectively.
Hager, E., Flagg, A. (2018). How Incarcerated Parents are Losing Their Children Forever. Retrieved from https://www.themarshallproject.org/2018/12/03/how-incarcerated-parents-are-losing-their-children-forever.	A report highlighting incarcerated mothers and fathers who have children placed in foster care process of regaining parental rights.	To provide examples of how incarcerated parents are losing their children.	Mothers and fathers who have a child placed in foster care because they are incarcerated are more likely to have their parental rights terminated than those who physically or sexually assault their kids. According to a Marshall Project analysis of approximately 3 million child-welfare cases nationally.
Huebner BM, DeJong C, Cobbina J. Women Coming Home: LongTerm Patterns of Recidivism. *Justice Quarterly* 2010;27:225–254.	Logistical and Survival analysis of data collected from a sample of 506 women released from prison in 1998 through May 2006.	To examine the long-term patterns of recidivism among a large, diverse sample of women released from prison in one state	The study found that women who are drug dependent, have less education, or have more extensive criminal histories are more likely to fail on parole and to recidivate more quickly during the eight-year follow-up period.
Jason LA, Salina D, Ram D. Oxford recovery housing: Length of stay correlated with improved outcomes for women previously involved with the criminal justice system. *Substance Abuse* 2016;37:248–254.	Randomized study of 200 women assigned to either the Oxford house recovery homes or usual care.	To examine the influence of recovery homes on a sample of former female substance-using women with criminal justice involvement.	Those with longer stays in the Oxford home had better outcomes in terms of alcohol and drug use, employment, and self-efficacy than those with shorter stays.
Lam H, Harcourt M. The Use of Criminal Record in Employment Decisions: The Rights of Ex-offenders, Employers and thePublic. *Journal of Business Ethics* 2003;47:237–252.	A review of legal approaches available for providing such protection by examining the diversity of approaches adopted in the federal and state jurisdictions of Australia.	To examines the need for legal protection of ex-offenders by limiting employer’s access to, and use of, information on criminal background.	The argument against accessing records state that ability to deny someone employment based on record is an unjustified extension of legal punishment and pushes them towards crime.
La Vigne N, Davies E, Palmer T, Halberstadt R. Release Planning for Successful Reentry A Guide for Corrections, Service Providers, and Community Groups. Urban Institute, editor. 2. 2008. Washington, DC, Urban Institute, Justice Policy Center. 10-2-2017.	A national survey of state correctional departments, a complimentary scan of practice, and a literature review on the topic of release planning.	To assist corrections agencies and their community partners in developing and improving their release planning procedures.	Corrections agencies must assess and incorporate an inmate’s strengths, weaknesses, and needs into one comprehensive document that the inmate can both understand and follow.
McCarty M, Falk G, Aussenberg RA, Carpenter DH. Drug Testing and Crime- Related Restrictions in TANF, SNAP, and Housing Assistance. *Journal of Drug Addiction*, Education, and Eradication 2012;8:71–98.	Report-describes the similarities & differences in federal policies governing drug & crime related restrictions in TANF, SNAP, & housing assistance programs.	To highlight a current set of crime- and drug-related restrictions in federal assistance programs inconsistencies.	There is an overall absence of evidence of the impact and effectiveness of crime- and drug-related restrictions in federal assistance programs. In part, the challenge is identifying the desired objectives of crime- related restrictions in federal assistance programs. Literature, however, does reveal how these policies become barriers for women on parole or probation and instead facilitate recidivism.
Metraux S, Culhane DP. Homeless Shelter Use and Re- incarceration Following Prison Release. *Criminology & Public Policy* 2004;3:139–160.	Survival analysis of time since prison release and history of residential instability.	To examine the incidence of and interrelationships between shelter use and re-incarceration among women released from prison.	Within two years of release, 11.4% of the study group was again imprisoned. Using survival analysis methods, time since prison release and history of residential instability were the most salient risk factors related to shelter use and shelter use increased the risk of subsequent re-incarceration.
Nargiso JE, Kuo CC, Zlotnick C, Johnson JE. Social Support Network Characteristics of Incarcerated Women with Co- Occurring Major Depressive and Substance Use Disorders. *Journal of Psychoactive Drugs* 2014;46:93–105.	Descriptive statistics and paired- tests were conducted on 60 incarcerated MDD- SUD women receiving in- prison substance use and depression treatments.	To characterize the women’s social networks, including the strength of support, network characteristics, and types of support provided as well as to determine what aspects of social support may be amenable to change during incarceration and post-release.	On average, women perceived they had supportive individuals in their lives, although more than a quarter of the sample could not identify any regular supporters in their network at baseline.
O’Brien P. Maximizing Success for Drug-Affected Women After Release from Prison. *Women & Criminal Justice* 2007;17:95–113.	Literature review and qualitative study of formerly detained or incarcerated drug- affected women.	To describe some of the correlates of drug-affected women and their involvement in the criminal justice system and findings from a study of drug-convicted African-American women who returned from prison to an economically disinvested community in Chicago.	Recommendations are suggested when working with formerly incarcerated women reentering the community: 1) a comprehensive and multidimensional assessment of psychological, social, and educational needs prior to release; 2) assistance with identifying family issues for family conferencing and negotiation; and 3) closer attention to job placement that enables women to gain income and gradual experience in the labor market.
Parsons ML, Warner-Robbins C. Factors That Support Women’s Successful Transition to the Community Following Jail/Prison. *Health Care for Women International* 2002;23:6–18.	A qualitative study that utilizes open- ended interview questions of women who participate in Welcome Home Ministries, a new community faith-based program for women released from jail/prison.	To describe factors that support women’s successful transition to the community following incarceration.	The role of support groups and their sisters in welcome home ministries, the nurse- chaplains jail visit and support, and the role of supportive friends (not former drug using friends) were additional key factors that help in successful transition.
Ramirez R. Reentry Consideration for Justice- Involved Women. 2016. The National Resource Center on Justice Involved Women. 11–29-2017.	NA	To document the critical differences— and by adopting gender-informed strategies shown by research to meet women’s unique needs—institutional corrections and community supervision agencies can maximize the success of women re-entering the community and improve the safety of both communities and correctional settings.	Key factors that have emerged in various women’s pathways to crime include experiences of abuse or trauma, poverty and marginalization, mental health disorders, substance abuse, and dysfunctional relationships.
Rogers E. Diversion Programs in America’s Criminal Justice System. 3–30. 2015. Washington DC, The Center for Prison Reform. 10-1-2017.	Review of diversion programs in 17 US states.	To examine the diversion program effectiveness on behaviorally correct lawbreakers, ensuring they do not offend again.	Jail diversion programs and other forms of alternative sentencing are an effective substitute for jail.
Salem BE, Nyamathi A, Idemundia F, Slaughter R, Ames M. At a Crossroads: Reentry Challenges and Healthcare Needs among Homeless Female Ex- Offenders. *J Forensic Nurs* 2013;9:14–22.	A qualitative study evaluating focus groups of 14 female ex- offenders enrolled in a residential drug treatment program in Southern California.	To understand the unique gendered experiences of homeless female ex- offenders, in the context of healthcare needs, types of health services sought, and gaps in order to help them achieve a smooth transition post-prison release.	Homeless female ex-offenders have a myriad of healthcare challenges, knowledge deficits, and barriers to moving forward in life, which necessitates strategies to prevent relapse.
Shinkfield AJ, Graffam J. Community Reintegration of Ex-Prisoners*. Int J Offender Ther Comp Criminol* 2009; 53:29–42.	Qualitative 79 prisoners (54 male & 25 female) were interviewed one month prior to release, 36 were interviewed one to four weeks post-release 19 three to four-month post- release.	To examine the multiple, complex, and dynamic nature of variables influencing successful reintegration by assessing the type and degree of change in reintegration variables over time.	Perceived physical health was better initially following the release. Housing stability was high over the post-release period.
The Lion Heart Foundation. Houses of Healing. Retrieved from https://lionheart.org/prison/state-by-state-listing-of-re-entry-programs-for-prisoners/.	A state-by-state listing of re- entry programs for prisoners.	A compiled a list of reentry programs below, listed by state, to help people connect with the services or contacts they might need.	The Lionheart Foundation’s Houses of Healing program has had a life-changing impact for thousands of the men and women across the country involved in the criminal justice system, providing them with the skills needed for successful reentry into the community.
Visher CA, Travis J. Transitions from Prison to Community: Understanding Individual Pathways. *Annu Rev Sociol* 2003;29:89–113 (Visher & Travis, 2003).	A review of literature on reentry failures.	To summarize what we know about the four specified dimensions and how they affect an individual’s transition from prison to community.	The review concludes with a call to the research community for interdisciplinary, multilevel, longitudinal studies of the processes of reintegration for former prisoners. Such research may illuminate many dimensions of social life, including the effects of recent social policies.
Walter RJ, Viglione J, Tillyer MS. One Strike to Second Chances: Using Criminal Backgrounds in Admission Decisions for Assisted Housing. *Housing Policy Debate* 2017;27:734–750.	Applies recidivism research to the use of criminal histories for assisted housing admission policies and procedures	This research examines several questions critical to assisting housing providers to address the new guidance from HUD.	Findings provide direction for housing providers on understanding recidivism risk rates, using useful lookback periods, considering risk and harm across crime types, and verifying rehabilitation and other evidence to design informed policies and procedures for using criminal records in admission decisions for assisted housing.
Warner-Robbins C, Parsons ML. Developing Peer Leaders and Reducing Recidivism Through Long-Term Participation in a Faith-Based Program: The Story of Welcome Home Ministries. *Alcoholism Treatment Quarterly* 2010; 28:293–305.	Welcome Home has provided service to more than 300 women per year who have been released from jail or prison into San Diego County communities.	To examine the effectiveness of welcome home ministries in assisting women through the change process and reduce recidivism.	To date, more than 80% of the women we have served have been able to sustain their recovery and avoid additional offenses requiring a return to jail or prison. Welcome Home has helped women go to college, embark on careers in drug and alcohol counseling or nursing, and reunite with their families.
Willging CE, Nicdao EG, Trott EM et al. Structural Inequality and Social Support for Women Prisoners Released to Rural Communities. *Women Crime Justice.* 2016; 26 (2): 145–164.	In-depth semi-structured interviews and focus groups with women prisoners in underserved rural communities.	To examine the return of women prisoners to underserved rural communities, while attending to the perspectives of their closest social supporters.	Rural women being released from prison and their closest social supporters, particularly family, appeared to internalize expectations that they take singular responsibility for their own wellbeing.
Worden, R. E., & McLean, S. J. (2018). Discretion and Diversion in Albany’s Lead Program. *Criminal Justice Policy Review*, *29* (6–7), 584–610.	Semi-structured interviews were administered to officers and surveys were conducted with officers.	To examine the exercise of officers’ discretion in making LEAD diversions by analyzing eligible incidents to estimate the effects of offense-, suspect-, and officer-related variables on discretionary decisions, and by analyzing semi structured interviews with officers.	Diverted arrests stemmed (with one exception) from four types of offenses: drug possession; theft (shoplifting); trespassing; and alcohol offenses (open container or public consumption). Only 77% of the LEAD participants had any criminal history.
Wolff N. Community reintegration of prisoners with mental illness: A social investment perspective. *Int J Law Psychiatry* 2005;28:43–58.	Profiles 2715 male special needs population in New Jersey prisons.	To describe behavioral health and criminal justice characteristics of 2715 male inmates with mental health problems, and identify the scope and nature of the public’s investment opportunity.	Approximately 67% were identified as having a serious mental illness. 26.4% were diagnosed with schizophrenia or other psychotic disorders; 41.1% with major depression, major mood disorder, or bipolar; 16.8% with depression, dysthymia, obsessive-compulsive disorder, PTSD; 12.8% with panic disorder, anxiety disorder, somatoform disorders, impulse control disorders, or ADD/ADHD; and 3.4% had an Axis II diagnosis only.
Zeoli AM, Rivera EA, Sullivan CM, Kubiak S “Post-separation abuse of women and their children: Boundary-setting and family court utilization among victimized mothers”: Erratum *J Fam Violence*. 2013 Aug 1; 28 (6): 547–560.	In-depth, qualitative interviews were conducted with 19 mothers who had divorced IPV-perpetrating husbands between one and three years prior. Participants were located through publicly available family court divorce records and interviews were examined using analytic induction.	To examines women’s responses to abuse committed by ex-husbands with whom they had undergone custody disputes.	Mothers often turned to family court for assistance in setting boundaries to keep children safe, but found that family court did not respond in ways they believed protected their children. Conversely, when women turned to the justice system for restraining orders or called the police for help against IPV, they generally found the justice system responsive.

**Table 4:** Domain Three: Healthcare Organizations Literature Review

Source	Study Description	Purpose	Results
Healthcare			
Bandara SN, Huskamp HA, Riedel LE et al. Leveraging The Affordable Care Act To Enroll Justice-Involved Populations In Medicaid: State And Local Efforts. *Health Aff (Millwood)* 2015;34:2044–2051.	A review of 64 programs operating in jails, prisons, or community probation and parole systems that enroll individuals during detention, incarceration, and the release process was conducted.	To describes four practices that have facilitated the Medicaid enrollment process: suspending instead of terminating Medicaid benefits upon incarceration, presuming that an individual is eligible for Medicaid before the process is completed, allowing enrollment during incarceration, and accepting alternative forms of identification for enrollment.	Seventy-seven percent of the programs are located in jails, and 56% use personnel from a public health or social service agencies.
Bracken, N., Hilliard, C., McCuller, W. J., & Harawa, N. T. (2015). Facilitators of HIV Medical Care Engagement Among Former Prisoners. *AIDS education and prevention: official publication of the International Society for AIDS Education*, *27* (6), 566–583.	Qualitative focus groups to understand factors that facilitated linkage to and retention in HIV care following their release.	To conduct seven focus groups with recently incarceration individuals in a California State prison to understand those factors that facilitated linkage to and retention in HIV care following their release.	Four main themes emerged from the analysis: 1) interpersonal relationships, 2) professional relationships, 3) coping strategies and resources, and 4) individual attitudes. Improving HIV-related outcomes among individuals after their release from prison requires strengthening supportive relationships, fostering the appropriate attitudes and skills, and ensuring access to resources that stabilize daily living and facilitate the process of accessing care.
Braithwaite RL, Treadwell HM, Arriola KRJ. Health Disparities and Incarcerated Women: A Population Ignored. *Am J Public Health* 2008;98: S173-S175.	Editorial: The explosion of female inmates over the last 3 decades.	To highlight a criminal justice system that was designed for men by men, rendering needs of women largely ignored.	Two-thirds of incarcerated women have children younger than 18 years old and women are more likely to be a single head of households- which make the family units & children collateral damage. Women tend to receive more severe citation despite not being near as violent as men.
Colbert AM, Sekula LK FAU Zoucha R, Zoucha RF, Cohen SM. Health care needs of women immediately post- incarceration: a mixed methods study. *Public Health Nurs.* 2013 Sep- Oct;30 (5):409–19.	A mixed methods study: (1) a quantitative survey; and (2) qualitative interviewers with 34 women post-release.	To examine the health status of women with a recent history of incarceration and explore if or how women were accessing health care resources at the time immediately following the release.	The major health issues identified by participants included specific health problems affected by incarceration, mental health needs, routine health promotion and maintenance, recovery from substance abuse as a major health concern, and social and environmental barriers to care.
Dias ER, da Silva Junior GB.Evidence-Based Medicine in judicial decisions concerning the right to healthcare. *Einstein (Sao Paulo)* 2016; 14:1–5.	A qualitative study of 19 Brazilian court decisions related to the right to health care taking into consideration.	To analyze, from the examination of decisions issued by Brazilian courts, how evidence-based medicine was applied and if it led to well founded decisions, searching the best scientific knowledge.	32% (6) were made in reaction to public authorities, 68% (13) were made relative to healthcare insurance plans. 18 of 19 of the decisions were favorable to Plaintiffs. Only 10 decisions demonstrated discussions regarding the suitability of the medication or procedure as per best scientific evidence.
Enard KR, Ganelin DM. Reducing Preventable Emergency Department Utilization and Costs by Using Community Health Workers as Patient Navigators. *J Healthcare Manag* 2013;58:412–428.	A nonequivalent comparison the group, quasi-experimental study design including pretest and posttest observations at 12 and 24 months for the intervention group and a nonrandomized control group with similar characteristics.	To examine a patient navigation program designed to promote appropriate primary care utilization and prevent or reduce Primary care- related emergency department use at Memorial Hermann Health System in Houston, Texas.	The patient navigation intervention was associated with decreased odds of returning to the ED among less frequent PCR-ED users.
Erlyana E, Fisher DG, Reynolds GL. Emergency room use after being released from incarceration. *Health Justice* 2014; 2:5 (Erlyana, Fisher, G., & Reynolds, 2014).	The Risk Behavior Assessment and Risk Behavior Follow-Up Assessment were administered to 1341 participants who were seeking STI testing and used the the emergency department in the last 3 months.	To provide insight into the associated costs of healthcare for previously incarcerated persons and the need for drug treatment during their incarceration.	31% used ED. Compared to those who did not use ED, those who did were more likely to have a history of incarceration (76% v 66%), a longer average time in jail, and ever-traded sex for drugs, & more likely to be opiate users.
Fox, A. D., Anderson, M. R., Bartlett, G., Valverde, J., Starrels, J. L., & Cunningham, C. O. (2014). Health outcomes and retention in care following release from prison for patients of an urban post-incarceration transitions clinic. *Journal of health care for the poor and underserved*, *25* (3), 1139–1152.	A retrospective cohort study that investigates care delivery and health outcomes for recently released prisoners.	To evaluate medical care delivery at an urban post-incarceration transitions clinic focusing on timely access to medical care, health outcomes, and retention in care for formerly incarcerated persons who were recently released from prison.	The median number of days between release from prison and the first medical visit was 10 days and 54% were seen within two weeks of release.
Hirsch MB. Health Care of Vulnerable Populations Covered by Medicare and Medicaid. *Health Care Finance Rev* 1994;15:1–5.	A discussion of articles published in this issue of the Health Care Financing Review, entitled “Health Care Needs of Vulnerable Population”.	To discuss articles cover the following vulnerable population subgroups: pregnant women and children, persons with AIDS, the disabled, and the elderly. Issues covered in this collection include: expenditures, demographic factors, Medicaid and Medicare policy, service use, medical procedures, and data collection	The collection of articles uses data from multiple sources and covers issues relevant to vulnerable population subgroups that are beneficiaries of the financing programs Health Care Finance Administers.
Kelly PJ, Hunter J, Daily EB, Ramaswamy M. Challenges to Pap Smear Follow-up among Women in the Criminal Justice System. *J Community Health* 2017;15–20	In-depth interviews with 44 women in the urban county jail.	To explore experiences with Pap tests and how they follow-up with abnormal results.	Women with criminal justice histories have numerous and complex challenges in following-up abnormal Pap test results, as well as other health problems. Four themes emerged: 1) Pap test abnormality; 2) unstable lives; 3) the structural challenges of money; and 4) competing demands.
Pager D. The Mark of a Criminal Record. *American Journal of Sociology* 2003;108:937–975 (Pager, 2003).	Experimental audit approach on the consequences of incarceration for the employment outcomes of black and white job seekers.	To formally test the degree to which a criminal record affects subsequent employment opportunities.	A criminal record presents a major barrier to employment, with important implications for racial disparities.
Ramaswamy M, Upadhyayula S, Chan KYC, Rhodes K, Leonardo A. Health Priorities among Women Recently Released from Jail. *American Journal of Health Behavior* 2015;39:222–231	Semi-structured interviews with 28 previously incarcerated women post-release.	To identify the priorities of women recently released from jail, and in particular, the context in which they set these priorities against other reentry concerns.	Three key themes emerged: 1) competing priorities after release from jail- children and employment, 2) health as a low priority- and barriers of transportation and money, and 3) context in which women used healthcare- indicated that health was a priority. 15.4% of women reported using ED for medical care.
Roth A, Fortenberry JD FAU Van Der Pol B, Van Der Pol BF et al. Court-based participatory research: collaborating with the justice system to enhance sexual health services for vulnerable women in the United States.	The mixed-methods study includes semi-structured interviews and focuses group discussions that were used to explore health-seeking behaviors, perceived gaps in services and components of court based screening program.	To examine a court-based program and how it facilitates justice-involved women to get sexually transmitted infections screening and other health services.	Community-based participatory research (CBPR) principals aided in research question development & equitable processes. Individual & socio-structural sources of health disparities considered.
Ryan J, Pagel L, Smali K. Connecting the Justice- Involved Population to Medicaid Coverage and Care: Findings from Three States. 2016. The Kaiser Commission on Medicaid and the Uninsured.	Telephone interviews conducted with a range of stakeholders in early 2016 to provide a brief overview of initiatives to connect the justice-involved population to Medicaid coverage and care in three states—Arizona, Connecticut and Massachusetts.	To examine programs and services that connect justice-involved women to Medicaid.	Supported by strong leadership, commitment, and close collaboration across agencies, the initiatives in these states have led to increased coverage, facilitated access to care, and contributed to gains in administrative efficiencies and state savings.
Springer SA. Improving Healthcare for Incarcerated Women. *J Womens Health (Larchmt)* 2010; 19:13–15.	Editorial of an article by Nijhawan et al.	To highlight an article by Nijhawan et al. that evaluated the important aspects of identifying preventive healthcare needs of incarcerated women.	Upon incarceration, important screening and prevention services should be offered universally to all prisoners, including immediate STD screening, cervical cancer screening with Pap smears, and breast cancer screening with mammograms.

## Abbreviations

CHDS: Community Health Delivery System
DCF: Department of Child and Family Services
ED: Emergency Departments
HUD: The Department of Housing and Urban Development
IOM: Institute of Medicine
LHD: Local Health Department
TANF: Temporary Assistance for Needy Families
